# Cross-Protocol Domain Gap in Internet of Things Intrusion and Anomaly Detection: An Empirical Internet Protocol-to-Bluetooth Low Energy Study of Domain-Adversarial Training

**DOI:** 10.3390/s26041184

**Published:** 2026-02-11

**Authors:** Hyejin Jin

**Affiliations:** School of Software, College of Computer Science, Kookmin University, Seoul 02707, Republic of Korea; hjjin@kookmin.ac.kr

**Keywords:** IoT security, intrusion detection, anomaly detection, unsupervised domain adaptation, domain-adversarial training, Bluetooth Low Energy, cross-protocol domain shift

## Abstract

**Highlights:**

**What are the main findings?**

- Cross-protocol IP → BLE transfer yields high seed-to-seed variability under label-free target conditions.
- Domain-adversarial training shows transient domain confusion; R3 (domain-aware checkpointing via domain-discriminator accuracy) improves target ROC-AUC without target labels, while classical ML baselines remain strong in this 14D setting.

**What are the implications of the main findings?**

- Random window-level splits can be optimistic; capture-wise/LOCO evaluation and operating-point audits (e.g., micro-FPR) are critical for deployment-faithful reporting.
- Monitoring domain-discriminator behavior (DomAcc, domain-discriminator accuracy) curves helps avoid misleading final-epoch conclusions in adversarial UDA.

**Abstract:**

Intrusion and anomaly detectors trained on Internet Protocol (IP) traffic are increasingly deployed in heterogeneous IoT environments where Bluetooth Low Energy (BLE) links coexist with IP networks. We quantify the cross-protocol domain gap in an IP → BLE transfer setting under unsupervised domain adaptation (UDA), where target labels are unavailable for training and model selection. Using 14 lightweight window-level statistics and leakage-aware splits, we benchmark classical baselines and alignment methods (CORAL and MMD) against domain-adversarial neural networks (DANNs). Under random window splits, DANNs can yield modest target gains but exhibit strong seed sensitivity and non-monotonic domain confusion. We propose R3, a domain-aware checkpoint rule that combines near-best source validation with domain discriminator accuracy as a proxy for alignment, improving the target ROC-AUC by ~+0.053 across three representative seeds and producing more consistent AP gains over 20 seeds. However, under a stricter capture-wise leave-one-capture-out (LOCO) protocol, UDA collapses to near-chance ranking and can underperform simple baselines, highlighting the risk of optimistic random splits. Finally, we show that transferring a source-tuned threshold can trigger unsafe operating points (micro-FPR = 1.0 on benign-only captures), motivating PR-based metrics and calibration/operating-point audits. We have released derived feature tables, split definitions, and scripts to support reproducibility under restricted raw data access.

## 1. Introduction

Internet of Things (IoT) deployments are increasingly heterogeneous: the same security analytics pipeline may be required to operate across multiple communication protocols and sensing stacks. In practice, intrusion and anomaly detectors are often trained on a single source domain and then deployed to a different target domain, where the feature distribution and traffic dynamics differ. This cross-protocol domain shift is a critical yet under-studied failure mode for IoT intrusion/anomaly detection, especially when target-domain labels are limited or unavailable. This perspective aligns with broader AI-IoT security and privacy challenges in advanced IoT services [1].

Motivating scenario: Many sensor-rich IoT systems use Bluetooth Low Energy (BLE) for low-power device-to-gateway communication and IP for backhaul. In practice, security analytics are frequently developed and tuned using IP network telemetry, but deployment at the edge (e.g., BLE gateways or embedded monitors) changes the observable traffic statistics due to protocol- and link-layer dynamics. This cross-protocol mismatch can induce substantial domain shift and degrade intrusion/anomaly detection, motivating principled cross-protocol adaptation.

We study a concrete cross-protocol transfer setting, IP → BLE, where the detector is trained on IP traffic features and evaluated on BLE traffic features. Our focus is on unsupervised domain adaptation (UDA): model selection and hyperparameter tuning are performed without target-domain training labels, and the target labels are used only for final evaluation.

Our contributions are as follows:

- We provide an empirical, reproducible evaluation of cross-protocol transfer (IP → BLE) using three randomized seeds for the main analyses, complemented by additional 20-seed repeats and paired statistical tests reported in the Appendix A and Appendix B/Supplementary Sections to quantify seed-to-seed instability.
- We benchmark classical distribution-alignment baselines (CORAL, correlation alignment; MMD, maximum mean discrepancy) and neural unsupervised domain adaptation (UDA) strategies, including domain-adversarial neural networks (DANNs) with a gradient reversal layer (GRL) and a control variant without the adversarial signal (noGRL).
- We complement performance metrics with domain-gap diagnostics and calibration/operating-point audits, and we propose domain-aware checkpoint selection (R3) without target labels, using the domain discriminator accuracy as a proxy for transient alignment and checkpoint sensitivity. Our contribution is primarily empirical and diagnostic: we do not introduce a new UDA algorithm but instead provide actionable findings for cross-protocol deployment.

The remainder of this study is organized as follows: Section 2 reviews related work; Section 3 describes the datasets, protocols, and methods; Section 4 presents the experimental results; Section 5 discusses practical implications and limitations; and Section 6 concludes the paper.

## 2. Related Work

### 2.1. IoT Intrusion and Anomaly Detection Under Distribution Shift

Learning-based IoT intrusion detection systems (IDSs) and anomaly detectors are often developed under a closed-world assumption: deployment traffic is drawn from the same distribution as the training data. In operational IoT, distribution shift is the norm—device firmware and protocol stacks evolve, deployments vary in topology and background services, and feature-extraction pipelines differ across platforms. Therefore, public IDS benchmarks (e.g., UNSW-NB15, CICIDS2017, Bot-IoT, TON_IoT, IoT-23, and Edge-IIoTset) exhibit heterogeneous benign traffic composition and attack implementations, which makes cross-dataset generalization difficult [2,3,4,5,6,7]. Recent work has quantified this effect in domain-specific settings (e.g., IoT vs. Internet of Medical Things (IoMT)), reporting large cross-dataset F1 drops when models trained on one dataset are tested on another [8].

Protocol heterogeneity further amplifies this shift. Moving from IP traffic to BLE changes framing, timing, and link-layer behavior, which can distort common flow features and induce a cross-protocol domain gap, even when the attack semantics are comparable. While BLE security procedures and threat surfaces are increasingly documented [9,10], BLE intrusion/anomaly detection remains relatively less explored and is often constrained by monitoring limitations (e.g., reliance on sniffers) and protocol dynamics such as channel hopping [11,12]. These characteristics motivate empirical cross-protocol studies that explicitly measure the domain gap and evaluate adaptation strategies under realistic constraints.

### 2.2. Unsupervised Domain Adaptation and Adversarial Alignment

Unsupervised domain adaptation (UDA) leverages labeled source data and unlabeled target data to learn representations that generalize to the target domain. Classical alignment includes moment matching with maximum mean discrepancy (MMD) [13] and covariance alignment (Deep CORAL) [14], while adversarial approaches such as DANNs optimize a feature extractor via gradient reversal to confuse a domain discriminator [15]. More recent UDA strategies include self-training/pseudo-labeling and contrastive representation learning objectives, but these often require additional confidence calibration or augmentation design; we use a DANN as a canonical adversarial baseline because its domain discriminator explicitly exposes domain-separability dynamics that we can audit under the target-unlabeled constraint. In network security, transfer learning has been explored to improve robustness across networks and datasets, including transfer learning-based IDS frameworks in IoT/5G settings [16,17] and domain-confusion architectures designed specifically for intrusion detection [18]. However, adversarial alignment can be unstable and sensitive to optimization and model selection choices, especially when target labels are unavailable for early stopping [15,19,20]. Our study isolates these dynamics in an IP → BLE shift and links them to measurable domain-gap diagnostics and epoch-wise behavior.

### 2.3. Thresholding, Calibration, and Imbalanced Evaluation in IDS

Intrusion detection commonly operates under class imbalance, which motivates reporting threshold-free metrics such as ROC-AUC, and, especially, average precision (AP, area under the precision–recall curve). Precision–recall curves are often more informative than ROC curves in skewed settings [21,22]. By contrast, F1-score and confusion-matrix-derived rates depend on a chosen decision threshold and can be misleading when posterior scores are poorly calibrated. Modern neural networks can be miscalibrated even in distribution [23], and calibration may further deteriorate under distribution shift, making threshold transfer from source to target brittle. To avoid optimistic bias in UDA, our main protocol selects thresholds only on labeled source validation data, and we complement thresholded metrics with PR curves, confusion matrices, and bootstrap confidence intervals for key results [24].

## 3. Materials and Methods

### 3.1. Datasets and Feature Construction

We use two preprocessed packet-table datasets representing distinct protocols: an IP-based dataset (source domain) and a BLE dataset (target domain). Raw packet captures are restricted due to privacy and security considerations; however, we release the derived, anonymized window-level feature tables and all scripts/tables needed to reproduce the reported metrics and figures (Supplementary File S1). For feature construction, we extract packet length and time from the preprocessed packet tables using the columns {IP: length = ‘http.content_length’, time = ‘http.time’, label = ‘is_malicious’; BLE: length = ‘frame.len’, time = ‘nordic_ble.packet_time’, label = ‘is_malicious’}. We segment each trace into non-overlapping windows (window size = 64 packets; stride = 64) and compute 14 lightweight statistical features (packet-length statistics, inter-arrival-time statistics, and ratio features). The binary label y is inherited from the dataset-provided maliciousness indicator and is used only for source-supervised learning and final target evaluation under the UDA protocol.

Sanitized provenance and labeling: The released packet tables originate from curated packet captures collected for IoT intrusion/anomaly detection experiments and are distributed in sanitized CSV form; raw pcap files are not released to prevent leakage of sensitive network identifiers. The packet tables are produced by an upstream preprocessing pipeline that anonymizes the original captures and provides a binary packet-level label (is_malicious) for each record. Although the concrete packet realizations differ across IP and BLE, the label is intended to encode the same high-level semantics (attack vs. benign) across protocols; accordingly, we treat this setting as label-aligned cross-protocol domain adaptation. Residual semantic shift due to protocol-specific packetization and timing cannot be fully ruled out and is treated as part of the cross-protocol domain gap studied here.

A structured provenance summary and a data-usage map are provided in Table A5 and Table A6.

To improve reproducibility under restricted raw data, we provide sanitized provenance metadata (windowing parameters and column mappings) and the full derived feature tables in Supplementary File S1 (‘features/feature_build_meta.json’, ‘features/*_stat_features.csv’).

As shown in Table 1, the target BLE domain has a higher positive-class prevalence (attack ratio 0.581) than the source IP domain (0.500), indicating the class-prior shift in addition to covariate/representation shift across protocols.

### 3.2. Experimental Protocol and Splits

We evaluate cross-protocol transfer from IP (source) to BLE (target). For each seed, the source domain is split into train/validation/test, while the target domain is split into validation/test. Target labels are not used for training, only for reporting final target-domain metrics. Table 2 summarizes the split sizes for the representative run (seed = 2026).

Active protocol (zero-activity filtering): We exclude zero-activity windows defined by nonzero_ratio = 0; this filter is applied to both domains without labels. Relative to the passive window set (Table 1), active filtering removes 384/15,625 (2.46%) IP windows and 324/3041 (10.65%) BLE windows. Retained window counts per split are reported in Supplementary File S1.

Leakage control: Windowing uses a non-overlapping configuration (stride equals window size), so no packet appears in multiple windows. Splits are performed after windowing; thus, packet-level overlap across train/validation/test is prevented by construction. Nevertheless, windows derived from the same capture/session may remain temporally correlated. Therefore, we interpret the reported results as window-level generalization and recommend group-wise splitting (e.g., by capture file or device/session ID) as a stricter protocol when such identifiers are available; see the Limitations subsection (Section 5, Discussion).

Randomization and seeds: We report mean ± std over three seeds (2024/2025/2026). Each seed initializes all random number generators (Python 3.10.12, NumPy 1.26.4, PyTorch 2.7.1+cu118), affecting the deterministic generation of source/target splits, model initialization, and mini-batch shuffling. Target labels are never used for training or model selection under the UDA protocol. Unless otherwise noted, main tables/figures report three seeds (2024/2025/2026); Appendix A.4 and Supplementary File S2 provide a 20-seed robustness analysis (seeds 2024–2043).

### 3.3. Compared Methods

We compare five methods implemented with the same backbone and optimization settings. All methods share a feature extractor and a label classifier; adaptation methods add an alignment loss term computed from source and target mini-batches.

Alignment hyperparameters (CORAL/MMD): For CORAL, we use a closed-form covariance alignment (no extra scaling term). For MMD-ERM, we use a scheduled scalar weight β(ep, step) with loss = L_cls + β(ep, step)·L_mmd, where EPOCHS = 20, WARMUP_EPOCHS = 3, β_max = 1.0. We set β(ep, step) = 0 for ep ≤ WARMUP_EPOCHS; otherwise, β(ep, step) = β_max·clip(*p*, 0, 1), with *p* = ((ep − WARMUP_EPOCHS − 1)·n_steps + step)/((EPOCHS − WARMUP_EPOCHS)·n_steps) (see Supplementary File S1).

- ERM (empirical risk minimization; source only): Trains only on labeled source data.
- CORAL-ERM (correlation alignment): Adds a covariance alignment loss between source and target feature representations [14].
- MMD-ERM: Adds a maximum mean discrepancy (MMD) loss to match source and target distributions in feature space [13].
- noGRL (lambda = 0): Uses the DANN architecture but disables the gradient reversal layer (GRL), serving as a control for the domain-classification head.
- DANN (domain-adversarial neural network; GRL): Domain-adversarial training with a gradient reversal layer (GRL) encourages domain-invariant features [15].

Objective functions and training procedures are summarized below to fix the optimization targets and improve reproducibility.

Notation: Let Ds be the labeled source (IP) set, and Dt be the unlabeled target (BLE) set. We use a feature extractor *G*(·), a label classifier *C*(·) that outputs class logits, and (when applicable) a domain discriminator *D*(·) that outputs domain logits. The domain label is d ∈ {0, 1} (d = 0 for source/IP, d = 1 for target/BLE).

(1) $$L_{cls}=E_{\left(x_{s},y_{s}\right)∼D_{s}}\left[CE\left(C\left(G\left(x_{s}\right)\right),y_{s}\right)\right]$$

(2) $$L_{CORAL}=\frac{1}{4k^{2}}{∥Cov\left(F_{s}\right)-Cov\left(F_{t}\right)∥}_{F}^{2}$$

(3) $$L_{MMD}=\frac{1}{n_{s}^{2}}\sum_{i,i\prime }K\left(f_{i}^{s},f_{i\prime }^{s}\right)+\frac{1}{n_{t}^{2}}\sum_{j,j\prime }K\left(f_{j}^{t},f_{j\prime }^{t}\right)-\frac{2}{n_{s}n_{t}}\sum_{i,j}K\left(f_{i}^{s},f_{j}^{t}\right)$$

(4) $$\underset{G,C}{min}\underset{D}{max}\;L_{cls}\left(G,C\right)-\lambda_{d}\;L_{dom}\left(G,D\right)$$

Objectives: ERM optimizes Equation (1) using labeled source samples only. CORAL-ERM and MMD-ERM add the alignment terms in Equations (2) and (3), respectively, computed from source and target mini-batches. The DANN optimizes the min–max objective in Equation (4) via a gradient reversal layer (GRL). noGRL shares the same architecture as the DANN but disables adversarial gradients to the feature extractor (i.e., GRL is removed). The training loop is summarized in Algorithm 1.
**Algorithm 1.** Unsupervised domain adaptation training for IP → BLE (ERM/CORAL/MMD/DANN/noGRL)  Input: Labeled source set Ds = {(xs, ys)}; unlabeled target set Dt = {xt}; feature extractor G; label head C; (optional) domain head D; training epochs E; batch size b; alignment weight β(ep, step) (for CORAL/MMD); adversarial weight λ (for GRL/DANN).  Output: selected model checkpoint (G, C) and evaluation logs.  1. Initialize parameters of G and C (and D if applicable).  2. For epoch = 1 to E:  2.1. Sample a labeled mini-batch (Xs, Ys) from Ds (source-train) and an unlabeled mini-batch Xt from Dt (target split).  2.2. Compute features Fs = G(Xs) and Ft = G(Xt).  $L_{cls}$ 2.3. Compute classification loss L_cls on (Xs, Ys) using Equation (1). $L_{cls}$  2.4. If CORAL: Compute alignment loss using Equation (2). If MMD: compute alignment loss using Equation (3). Otherwise, set alignment loss to 0.  2.5. If DANN or noGRL: Compute domain loss *L_dom_* on (Xs, Xt) using Equation (4), update D to minimize *L_dom_*. For DANN, update G via the GRL with weight *λ* to maximize *L_dom_*; for noGRL, update G without the GRL signal (*λ* = 0).  2.6. Update (G, C) using source labels only; do not use any target labels during training or checkpoint selection (UDA protocol).  2.7. Evaluate on the source validation set; save a checkpoint if it improves the selection criterion (source-val ROC-AUC; tie-breaker: source-val AP; then earliest epoch).  3. Return the selected checkpoint. Optional diagnostic: record the epoch where domain accuracy is closest to 0.5 as epoch * for analysis.  The asterisk (*) denotes the diagnostic epoch (the epoch where domain accuracy is closest to 0.5).

### 3.4. Evaluation Metrics and Statistical Validation

We report target-domain receiver operating characteristic area under the curve (ROC-AUC) and average precision (AP); additionally, we provide an F1 score for a fixed decision threshold. To quantify uncertainty on a challenging split, we report bootstrap 95% confidence intervals on seed = 2026 by resampling target test windows with replacement.

In addition to performance metrics, we compute diagnostic measures of distribution shift between source and target representations. Specifically, we report the maximum mean discrepancy (MMD) [13] and sliced Wasserstein distance (SWD) [25] before and after adaptation (seed = 2026).

Decision thresholding: For threshold-dependent metrics (precision/recall/F1 and confusion matrices), we select a decision threshold, $\tau^{*}$, by maximizing the attack-class F1 on the labeled source validation split and then apply the same $\tau^{*}$ to BLE target test predictions. This protocol avoids target-label leakage, which is essential in UDA. Because cross-protocol shift can induce score miscalibration, $\tau^{*}$ may not transfer optimally; therefore, we treat AP/PR curves as primary indicators of detection quality under imbalance [21,22] and provide thresholded error analysis in Appendix A (Table A1).

Concrete mitigation for threshold transfer: Beyond auditing the source F1 threshold, τ*, we also compute a conservative source-validation threshold, τ (FPR = 1%), that achieves a 1% false-positive rate on source negatives and transfer it unchanged to BLE (see the calibration audit results in Section 4.6). While cross-protocol shift can still distort score calibration (and thus cannot guarantee a fixed target FPR), this provides an explicit risk-limiting knob and turns threshold transfer from a passive observation into a concrete audit + baseline mitigation under the target-unlabeled constraint.

Calibration considerations: Post hoc calibration techniques such as temperature scaling can improve in-distribution probability calibration [23]. However, calibration itself can drift across domains, and thus, source-only calibration does not guarantee well-calibrated target scores. Therefore, we report both threshold-free metrics (ROC-AUC and AP) and thresholded summaries, and we interpret F1 in conjunction with PR curves and confusion matrices rather than as a standalone indicator.

### 3.5. Implementation Details, Checkpoint Selection, and Reproducibility

Software/hardware: Experiments were run with Python 3.10.12 and PyTorch 2.7.1+cu118 (CUDA 11.8) on a single NVIDIA A100 80 GB GPU; NumPy 1.26.4, pandas 2.3.3, and scikit-learn 1.7.2 were used for preprocessing and evaluation. A complete environment snapshot is provided in Supplementary File S1. Key architecture and training settings are summarized in Table 3.

Model specification: All methods use the same multilayer perceptron (MLP) backbone on 14 statistical features. The feature extractor G is a 2-layer MLP (14 → 128 → 128 with ReLU); the label head C is a linear layer (128 → 2 logits); and for DANN/noGRL, the domain head D is a 2-layer MLP (128 → 64 → 2 logits).

GRL schedule: The DANN uses a 3-epoch warm-up ($\lambda_{d}$ = 0 for epochs 1–3), followed by a standard monotone schedule that increases the GRL weight toward 1 by the final epoch. The exact per-epoch $\lambda_{d}$ values are provided in ‘logs/dann_train_history.csv’ in Supplementary File S1.

Checkpoint selection: To avoid target-label leakage in UDA, the default ‘best checkpoint’ for each method is selected solely using labeled source-validation performance (primary: max source-val ROC-AUC; tie-breakers: source-val AP, and then earliest epoch). For DANN/GRL, we additionally evaluate R3 (Section 3.6), a domain-aware selection rule that uses only domain labels (source vs. target) to prefer checkpoints with maximal domain confusion (DomAcc near 0.5), without using target class labels.

Baseline fairness (classical ML): Classical baselines (LogReg/RF/XGB) are trained and configured without using any target-domain class labels. Any hyperparameter choices are fixed a priori or selected using labeled source-only validation; target samples are not used for tuning or selection. For thresholded audits, the decision threshold τ* is chosen based on source validation and transferred unchanged to the target, consistent with the UDA constraint.

Reproducibility package: Supplementary File S1 includes derived feature tables (IP/BLE), a split summary and seed list, trained checkpoints, training logs, and scripts to regenerate the reported tables and figures. This enables independent verification of the reported metrics without requiring access to restricted raw packet traces.

### 3.6. Domain-Aware Checkpoint Selection (R3)

Default checkpoint selection in UDA relies on labeled source validation only. For adversarial alignment, we additionally consider a domain-aware rule (R3): among near-best source-validation epochs, we select the checkpoint whose domain discriminator accuracy (DomAcc) is closest to chance (0.5). We define near-best epochs as those whose source-validation ROC-AUC is within δ of the best epoch, i.e., AUC_src_val(e) ≥ max_{e′} AUC_src_val(e′) − δ (δ = 0.001 in our experiments). This tolerance safeguards against selecting clearly undertrained/overfit epochs; when the source-validation ROC-AUC is nearly flat, R3 effectively reduces to selecting the epoch with DomAcc closest to 0.5. DomAcc is computed on a balanced domain-validation set by subsampling equal numbers from the source-validation split and the unlabeled target split (not the target test set) at each epoch; therefore, chance corresponds to 0.5, even when the two splits differ in size. Oracle epochs (maximizing target-test ROC-AUC) are reported only as an analysis upper bound. R3 never uses target class labels; it uses only domain labels (source vs. target). DomAcc is computed on unlabeled target data reserved for checkpointing (not on the target test set). To further reduce selection bias, we describe a stricter target-unlabeled holdout protocol in Supplementary File S1 (split definitions) and discuss its implications in Section 5.

## 4. Results

### 4.1. Cross-Protocol Target Performance

**Figure 1:**
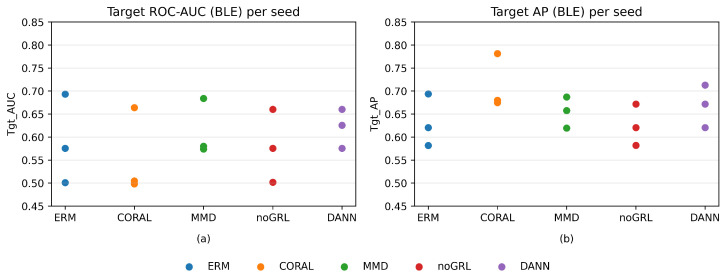
Per seed target BLE performance for all methods (seeds 2024/2025/2026). ROC-AUC: receiver operating characteristic area under the curve; AP: average precision. Points show each seed; this visualization complements Table 4 (mean ± std) and highlights seed-to-seed variability.

As summarized in Table 4 and visualized in Figure 1, DANN (GRL) achieves the best mean target ROC-AUC among the neural UDA strategies, while CORAL-ERM attains the highest mean target AP. For this low-dimensional (14D) feature space, classical baselines are competitive on the random window split: logistic regression (LogReg) achieves target AUC/AP 0.683 ± 0.027/0.756 ± 0.078, random forest (RF) achieves 0.758 ± 0.051/0.770 ± 0.062, and XGBoost (XGB) achieves 0.711 ± 0.013/0.706 ± 0.009. Our aim is not to claim that deep UDA dominates classical ML in this 14D setting but to characterize cross-protocol failure modes and stabilize label-free model selection under UDA constraints. Finally, these random window-split results should be interpreted as optimistic upper bounds for deployment; Section 4.7 reports leakage-controlled leave-one-capture-out evaluation. Notably, an apparently high F1@$\tau^{*}$ for ERM/noGRL can correspond to a degenerate all-positive operating point under threshold transfer, which is operationally unsafe despite benign-looking summary scores (Table A1).

### 4.2. Bootstrap Confidence Intervals on Seed 2026

To validate the target-domain gains under a challenging split (seed = 2026), Table 5 reports bootstrap 95% confidence intervals. On this split, ERM and noGRL yield near-chance ROC-AUC, whereas DANN (GRL) provides a substantial AUC improvement. The paired bootstrap additionally yields ΔAUC = 0.124 [0.096, 0.153] and ΔAP = 0.131 [0.106, 0.157] (DANN−ERM), confirming a statistically meaningful improvement on seed = 2026.

### 4.3. Unified Diagnostic Workflow for Cross-Protocol Domain Shift

To make the diagnostics in this section directly actionable under the target-unlabeled UDA constraint, we consolidate them into a deployment-oriented workflow (Table 6) that maps each proxy signal to a concrete modeling or reporting decision. This unified view aligns with broader risk auditing perspectives in safety-critical IoT/cyber-physical systems, where vulnerability assessment and defense should be accompanied by clear operational guidance [26,27]. The workflow is instantiated below with quantitative divergence proxies (Table 7 and Table 8) and a semantic-shift screen (Table 9).

Step 2 of Table 6 reports global representation-gap proxies. In the representative split (seed = 2026), SWD decreases after adaptation, whereas RBF-kernel MMD increases (Table 7), indicating that conclusions about ‘alignment’ can be metric-dependent. Given that kernel MMD is bandwidth-sensitive, Table 8 provides an RBF σ sweep to contextualize the reported value and avoid over-interpreting a single kernel setting.

Step 3 of Table 6 complements global discrepancies with a within-class semantic shift check: class-conditional Kolmogorov–Smirnov (KS) tests compare marginal feature distributions across domains within each label. Table 9 lists the most shifted features, helping explain why improvements in an aggregate discrepancy metric do not necessarily translate into deployment-faithful target performance.

### 4.4. Representation Alignment and Training Dynamics

**Figure 2:**
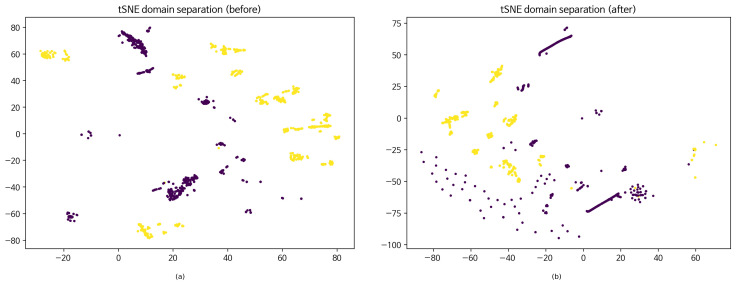
t-distributed stochastic neighbor embedding (t-SNE) visualization of source (IP) and target (BLE) embeddings (seed = 2026) obtained from the 128-d feature extractor: (**a**) before adaptation (ERM, source only) and (**b**) after domain-adversarial training (DANN/GRL). t-SNE parameters (scikit-learn): perplexity = 30, init = pca, learning_rate = auto, n_iter = 1000, and random_state = 2026. Points are colored by domain (source IP vs. target BLE), with two colors (purple and yellow) indicating the two domains.

**Figure 3:**
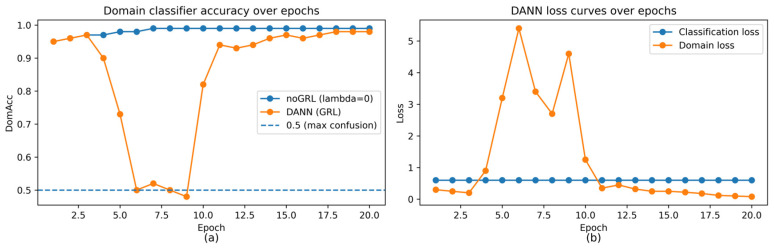
Domain discriminator behavior during domain-adversarial training (seed = 2026). (**a**) Domain classifier accuracy (DomAcc) over epochs computed on a balanced domain-validation set (equal source-validation and unlabeled target samples): 1.0 indicates perfect domain separability (no alignment), while 0.5 indicates maximal domain confusion (alignment). (**b**) DANN training losses over epochs (source classification loss and domain loss). The transient confusion phase motivates domain-aware checkpointing; R3 selects a ‘star’ checkpoint using DomAcc among near-best source-validation epochs (seed = 2026; star epoch: 20; Table 10).

Figure 2 visualizes the learned feature space using t-distributed stochastic neighbor embedding (t-SNE) before and after adaptation (panels (a) and (b), respectively). Figure 3 summarizes the key DANN training dynamics used by R3: domain classifier accuracy (DomAcc; panel (a)) and the corresponding loss curves (panel (b)).

To probe target-domain failure modes beyond aggregate scores, we compute a simple feature-level error analysis for the DANN on the BLE target test set. For each standardized feature, we compute the mean value within the four confusion groups (TP/FN/TN/FP) and rank features by |Δ(FN − TP)|, i.e., the magnitude of the separation between missed attacks (FN) and correctly detected attacks (TP). The top contributors (Figure 5) are dominated by packet-length dispersion statistics (e.g., pkt_len_mean, pkt_len_min, pkt_len_std) and timing variability (e.g., iat_std), suggesting that protocol-dependent burstiness and length distributions are key sources of cross-protocol errors.

**Figure 4:**
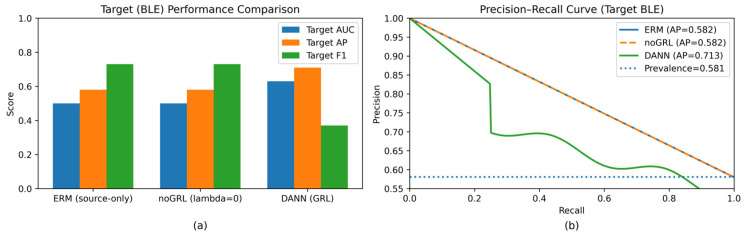
Target BLE performance summary across methods (seed = 2026): (**a**) metric summary (ROC-AUC/AP/F1); (**b**) target BLE precision–recall curve.

Figure 3b reports DANN loss curves, complementing the domain accuracy dynamics in Figure 3a. Figure 4 summarizes target BLE performance across methods (seed = 2026), and Figure 4b provides the corresponding precision–recall curve.

**Figure 5:**
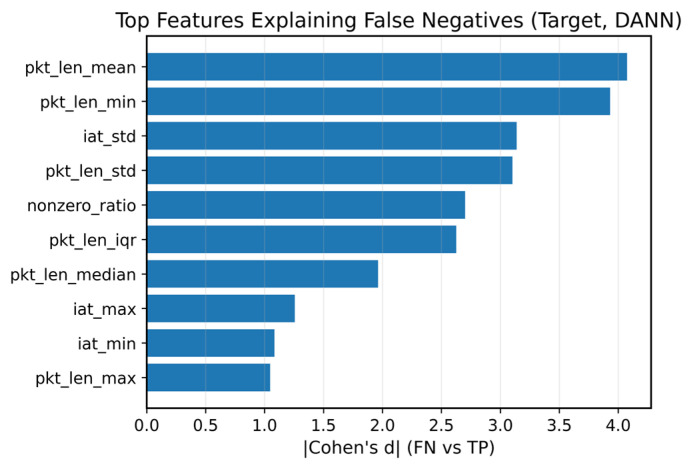
Top-10 feature-level error contributors for DANN on the BLE target test set (seed = 2026). Features are ranked by |(FN−TP)|, the absolute difference between the mean standardized feature value of false negatives (FN) and true positives (TP) on the target test set. Values are computed after applying the source-fitted standardization (StandardScaler).

**Figure 6:**
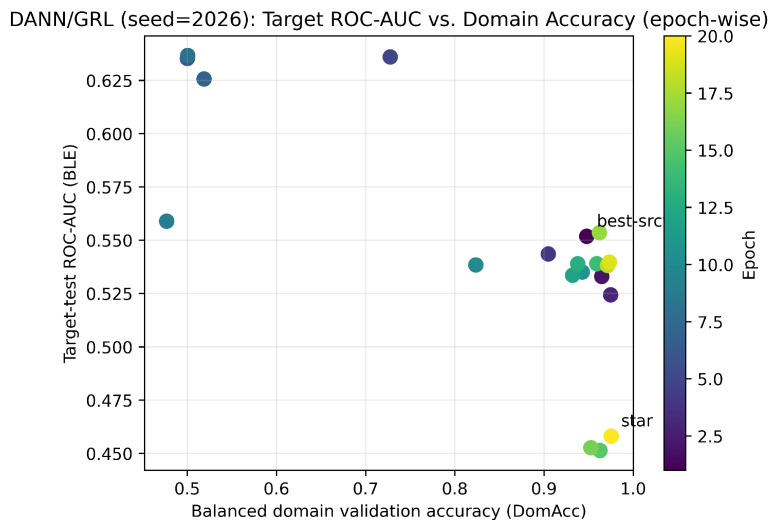
Epoch-wise target-test ROC-AUC versus balanced domain accuracy (DomAcc) for DANN/GRL (seed = 2026). Lower DomAcc values (closer to 0.5) indicate stronger domain confusion on the balanced domain-validation set.

**Figure 7:**
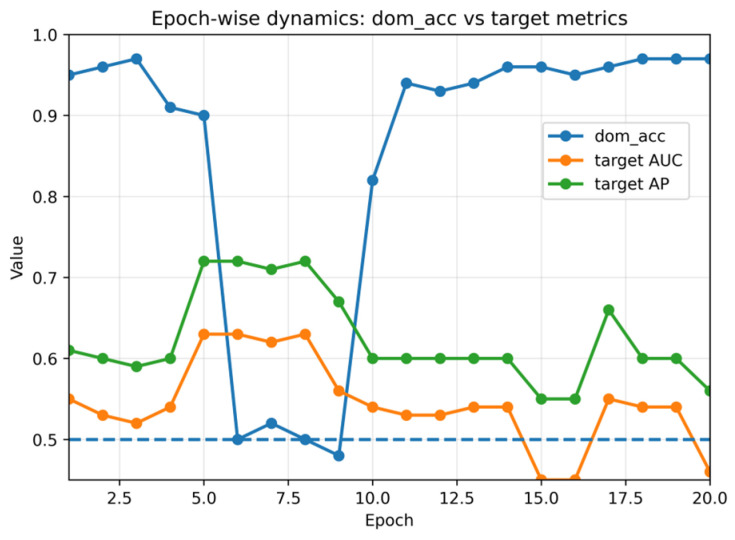
Training dynamics for DANN/GRL (seed = 2026): source-validation ROC-AUC, target-test ROC-AUC, and balanced DomAcc across epochs. DomAcc approaching 0.5 indicates transient domain confusion; DomAcc near 1.0 indicates that the domains remain separable. The dashed horizontal line indicates the chance-level balanced domain accuracy (DomAcc = 0.5).

Figure 3 summarizes domain-discriminator behavior and DANN training losses (seed = 2026). Figure 4 reports target BLE performance across methods (seed = 2026): Figure 4a shows the metric summary, and Figure 4b shows the precision–recall curve. Figure 5 provides feature-level error contributors for DANN on the target test set, while Figure 6 and Figure 7 relate target ranking performance to transient domain confusion and training dynamics via DomAcc.

### 4.5. Evaluation of Domain-Aware Checkpoint Selection (R3)

**Figure 8:**
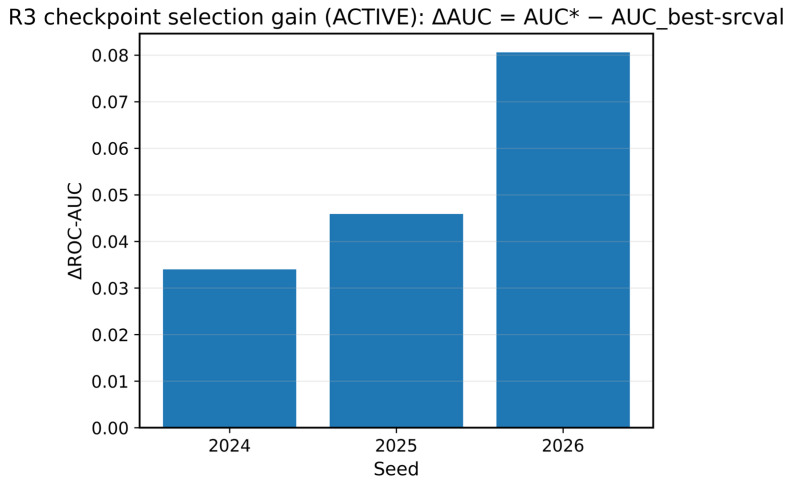
R3 gains in target-test ROC-AUC (active): per-seed improvement in target-test ROC-AUC for the domain-aware checkpoint selected by R3 (AUC*) relative to the default-best checkpoint (AUC_best); ΔROC-AUC = AUC* − AUC_best. The asterisk (*) is used only to denote the R3-selected domain-aware checkpoint (not a statistical significance marker).

**Figure 9:**
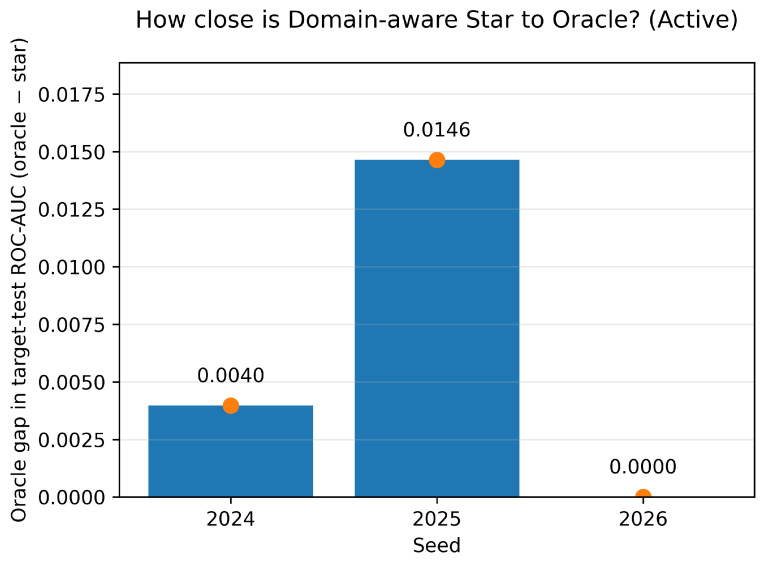
Oracle gap of domain-aware star (active): per seed oracle gap in target-test ROC-AUC (oracle—star). Oracle is computed post hoc and is used only as an analysis upper bound.

**Figure 10:**
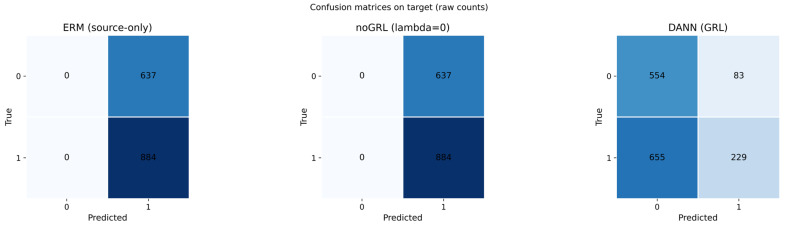
Threshold-transfer failure case on the BLE target test set (seed = 2026). Confusion matrices (raw counts) are shown for ERM (source only), noGRL (lambda = 0), and DANN (GRL), illustrating that ERM/noGRL can collapse to near all-positive predictions under τ* transfer, while DANN mitigates this failure mode. The color intensity indicates the magnitude of the raw counts in each confusion-matrix cell (darker blue = larger count).

We propose R3 (risk rank by confusion), a label-free checkpoint selection heuristic for UDA that leverages the domain discriminator. Specifically, we first identify a set of near-best epochs whose source-validation ROC-AUC is within δ of the best value (δ = 0.001 unless noted) and then choose the epoch whose balanced domain accuracy (DomAcc; Figure 3a) is closest to 0.5. The intuition is that a fully confused discriminator indicates stronger domain invariance, which can correlate with improved target performance under domain shift. The results are summarized in Table 10 and Table 11 and Figure 8, Figure 9 and Figure 10.

Given that R3 selects checkpoints based on transient domain confusion (DomAcc close to 0.5) rather than directly optimizing precision–recall ranking, modest AP decreases can occur even when ROC-AUC improves. In practice, the preferred checkpoint should reflect the operational objective (ROC-oriented ranking vs. PR-oriented early warning) and the chosen thresholding strategy under the prior shift.

Supplementary analyses further examine (i) the sensitivity of R3 to δ (Table S1). All split definitions and scripts required to reproduce these analyses are included in Supplementary File S1.

We summarize domain-aware checkpoint selection (R3) under the Active protocol and report a per-seed comparison between the default best (selected by source-validation ROC-AUC) and domain-aware star (selected by DomAcc closest to 0.5 among near-best epochs) in Table 10.

We report an oracle upper-bound analysis for checkpoint selection under the active protocol in Table 11, where the oracle epoch is defined post hoc as the checkpoint maximizing the target test ROC-AUC.

As shown in Figure 9, the oracle gap quantifies the remaining headroom of the domain-aware star relative to the oracle (oracle ROC-AUC − star ROC-AUC); the oracle is computed post hoc and used only as an upper bound for analysis.

### 4.6. Threshold Transfer and Operating-Point Sensitivity

Although AUC and AP are threshold-free, the reviewers requested that we evaluate a direct threshold-transfer scenario. In the active protocol, we compute the optimal decision threshold, τ*, on the source validation split (maximizing F1) and then apply the same τ* without recalibration to the target test split. We audit (i) the F1 drop under τ* transfer (Table 12) and (ii) calibration behavior (reliability diagrams in Figure 12). Figure 10 shows a representative threshold-transfer failure case on the BLE target (seed = 2026).

### 4.7. Extended Evaluation: Leakage-Controlled Evaluation and Operating-Point Analysis

To approximate deployment-faithful conditions where a detector is evaluated on an entirely unseen BLE capture, we additionally report a leakage-controlled leave-one-capture-out (LOCO) evaluation. Windows are generated with a non-overlapping scheme (window size = 64 packets, stride = 64) from preprocessed packet-level tables. For the source (IP) domain, we construct a mixed time-block group identifier (group_id_mixed) by accumulating consecutive windows in chronological order until both classes appear within each group, yielding 16 mixed groups (one-class group ratio = 0). For the target (BLE) domain, we define capture groups (ble_cap_k) using capture IDs. The LOCO protocol holds out an entire mixed-class BLE capture (ble_cap_2) for testing, while ble_cap_0/1 (benign-only) are used to audit operating-point behavior under purely benign deployments.

**Figure 11:**
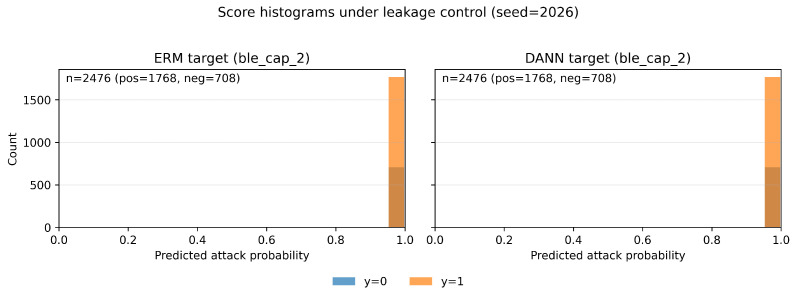
Target score histograms on the mixed-class BLE capture group (ble_cap_2) under leakage control (seed = 2026): ERM (**left**) vs. DANN (**right**).

Figure 11 shows target score histograms on the mixed-class BLE capture group (ble_cap_2) under leakage control (seed = 2026): ERM (left) vs. DANN (right).

**Figure 12:**
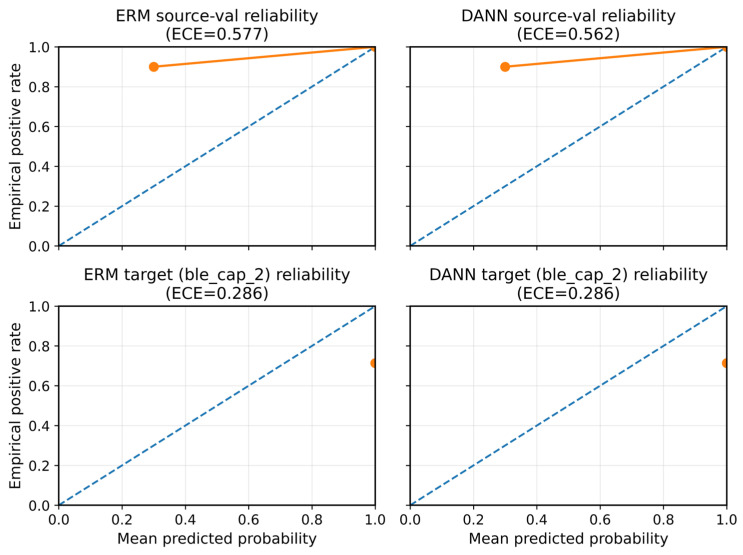
Reliability diagrams (calibration curves) for ERM and DANN under leakage-controlled evaluation. The solid curve shows the empirical calibration (fraction of positives) in each probability bin, and the dashed diagonal line indicates perfect calibration (y = x); deviations from the diagonal indicate miscalibration under domain shift.

Notably, on the only mixed-class capture (ble_cap_2), deep UDA does not improve ranking over ERM, and both the DANN and noGRL can even underperform a simple logistic regression baseline (Table 13; Figure 11 and Figure 12). For the two benign-only captures (ble_cap_0 and ble_cap_1), ROC-AUC and AP are undefined; therefore, we report micro-FPR at a source-calibrated operating point (FPR = 1% on source) and observe that the false-positive burden can be severe in a multi-capture LOCO analysis (Table 12). Consistent with Table 12, Figure 12 further illustrates that domain-adversarial training can improve calibration on the held-out mixed-class capture, although ranking performance remains near chance.

Given that two BLE capture groups (ble_cap_0 and ble_cap_1) contain only negative samples (pos_ratio = 0), ROC-AUC and average precision are undefined for those groups. Accordingly, we report ROC-AUC/AP only on the mixed-class capture group (ble_cap_2) and complement them with operating-point metrics (false positive rate and specificity) aggregated across all capture groups. Given that the positive prevalence in ble_cap_2 is high (pos_ratio = 0.714), AP should be interpreted relative to this prior; therefore, Table 13 includes AP-lift (AP − pos_ratio) to quantify improvement over a prevalence-only baseline. In the worst case, this corresponds to micro-FPR = 1.0 on benign-only captures (Table 12).

## 5. Discussion

Our results show that domain-adversarial training can improve cross-protocol target AUC, but the training dynamics are not monotonic. In particular, DomAcc_last remains high for DANN/GRL (0.973 ± 0.002), indicating that domain-discriminative information persists at convergence. The epoch-wise analysis suggests that the period of strongest domain confusion (domain accuracy near 0.5) coincides with peak target AUC/AP and that later epochs may overfit to domain-specific cues. As an exploratory check under the random-window setting (and not a like-for-like comparison with the capture-wise protocol summarized in Table A4), we evaluate self-training with pseudo-labeling over 20 seeds (Supplementary Tables S2–S5; Figures S1–S4). Across 20 seeds, the AUROC change is modest and not statistically significant (Wilcoxon *p* = 0.231), while AP improves modestly and is statistically significant (Wilcoxon *p* = 0.024); the paired Wilcoxon test results are provided in Table S3; benign-only micro-FPR remains degenerate under τ_F1 (micro-FPR = 1.0) but is reduced under a source threshold calibrated at FPR = 1% (Table S4). We report these results solely to document consideration of a representative recent UDA strategy; our main conclusions and key comparisons are based on the capture-wise protocol and operating-point audits (Table A4 and Table 12, and Figure 10, Figure 11 and Figure 12).

Why classical ML can outperform neural UDA in a 14D feature space: In our setting, the 14 window-level statistics are already a compact, engineered representation; the remaining learning problem is primarily a low-dimensional decision function rather than representation learning from raw packet sequences. Therefore, tree ensembles (RF/XGB) and regularized linear models can exploit non-linear feature interactions or stable convex optimization with limited risk of overfitting, whereas adversarial objectives in a DANN introduce an additional minimax optimization that is sensitive to learning rates, initialization, and discriminator capacity. Moreover, forcing domain confusion can attenuate class-discriminative cues when the feature space offers limited degrees of freedom, which helps explain why classical models can be competitive—or even superior—on the random window split (Table 4). We include this discussion to avoid over-interpreting the results as evidence against deep learning in general; with richer representations and higher-capacity sequence models, the relative ordering may differ.

Interpreting domain-gap metrics: In the representative split (seed = 2026), SWD decreases after DANN (66.115 → 40.679), whereas MMD increases (0.579 → 0.669). This is not necessarily contradictory, because MMD and SWD capture different facets of distribution mismatch. MMD is kernel-based and can be sensitive to the kernel choice/bandwidth, effectively emphasizing certain moment/higher-order discrepancies in an RKHS [13], while SWD approximates optimal transport by averaging 1D Wasserstein distances over random projections [25]. Adversarial training can reduce discrepancies along many projections (lower SWD) while still increasing kernel-based discrepancy if alignment changes higher-order statistics or creates sharper modes. Accordingly, we avoid treating any single domain-gap metric as a definitive “alignment score” and interpret gap diagnostics jointly with target performance and training dynamics. Practically, this implies that early stopping or checkpoint selection based on a single discrepancy (MMD or SWD alone) can be misleading; combining discrepancy diagnostics with DomAcc curves is safer in unlabeled target settings. Consistent with this sensitivity, our σ sweep (Table 8) shows that the magnitude of MMD^2^ varies substantially across bandwidths, underscoring that conclusions based on a single kernel setting can be unstable.

The LOCO collapse likely reflects a mixture of capture-dependent artifacts and genuine semantic/label shifts across captures. Given that the BLE domain exhibits both class-prior shift (Table 1) and score miscalibration under threshold transfer (Section 4.6), label-shift and calibration effects can amplify even modest representation mismatch. In addition, LOCO removes the within-capture temporal correlation that can make random window splits optimistic, thereby exposing capture-specific characteristics (e.g., device mix, channel conditions, and capture setup) that are not represented in training. While our current 14D statistics cannot fully disentangle these factors, the combined evidence from discrepancy metrics (Table 7), domain-discriminator dynamics (Figure 3), and operating-point failures (Table 12 and Figure 10) supports reporting capture-wise evaluation whenever possible. This interpretation is further supported by the class-conditional KS diagnostic (Table 9), which reveals substantial within-class distribution differences between IP and BLE for multiple packet-length and inter-arrival-time statistics.

These findings motivate two practical recommendations for cross-protocol IoT intrusion/anomaly detection: (i) monitor both unlabeled target split proxies and domain-classifier behavior during training and (ii) consider domain-aware checkpointing strategies, such as selecting checkpoints when the domain classifier is closest to chance or when the correlation between domain accuracy and target metrics becomes unfavorable.

Relation to unsupervised model selection in UDA: Model selection without target class labels is a recognized challenge in UDA, and prior work has proposed proxy criteria such as Deep Embedded Validation (DEV) for deep UDA model selection and Soft Neighborhood Density (SND) for unsupervised validation of domain adaptation; recent benchmarks further highlight that reliable unsupervised selection remains difficult across datasets and methods [28,29,30]. R3 is complementary: it leverages the domain discriminator already present in adversarial UDA and requires only domain labels (source vs. target) and a small tolerance, δ, for near-best source performance. Its main limitation is that it applies only when the domain discriminator is trained and informative (e.g., if DomAcc saturates near 1.0 throughout training, R3 reduces to source-only selection).

$\tau^{*}$ Threshold sensitivity and imbalance: Our analysis also highlights an evaluation pitfall that is common in IoT IDS under domain shift. At seed = 2026, ERM and noGRL produce near all-positive predictions at the source-selected threshold $\tau^{*}$, yielding perfect attack recall but zero true negatives on the BLE target (Table A1). This behavior inflates attack-class F1 while failing to discriminate benign traffic, and it is consistent with near-random ROC-AUC. Therefore, we emphasize AP/PR curves as primary in imbalanced regimes [21,22] and recommend explicitly reporting threshold-selection rules and calibration limitations under cross-protocol deployment [23]. Figure 10 (Section 4.6) visualizes this failure mode for seed = 2026, and Table A1 provides split-level operating-point audits.

Limitations: This study focuses on a single cross-protocol pair (IP → BLE) and a compact 14-feature family to isolate the domain-gap mechanism. We chose this lightweight, protocol-agnostic statistical representation because it is compatible with resource-constrained IoT deployments and enables apples-to-apples cross-protocol evaluation without relying on raw packet payloads. Nevertheless, absolute performance levels may differ under richer sequence/flow representations and higher-capacity architectures; our primary claims target diagnostic behaviors (evaluation optimism, checkpoint sensitivity, and threshold-transfer brittleness) rather than state-of-the-art accuracy. Although non-overlapping windowing prevents packet-level overlap across splits, stricter group-wise splits (e.g., by capture file or device/session ID) would further reduce potential temporal correlation; future work will include such protocols when group identifiers can be released. In addition, because the feature space is low-dimensional, classical ML baselines (e.g., logistic regression, SVM, random forests, and gradient boosting) are valuable for establishing whether deep UDA is necessary; we provide scripts and the derived feature tables in Supplementary File S1 to facilitate these comparisons. To partially broaden protocol coverage without changing the main scope, we provide an addendum evaluation on IP → Zigbee in Appendix B and Supplementary File S3; gains are less consistent in this secondary pair, reinforcing the need for broader protocol coverage. Finally, richer feature families and calibrated decision making under prior shift remain important directions for deployment-faithful cross-protocol IDS. Future work should validate these findings on additional protocol pairs and richer representations and explore adaptation objectives or training schedules that maintain domain confusion more stably toward convergence. Future work should also consider additional divergence diagnostics (e.g., proxy A-distance/C-distance or JS-based measures) to better separate semantic shift from capture artifacts. For additional optional diagnostic schematics and exploratory analyses that support reviewer discussion (e.g., PAD/MCD), see Supplementary File S4.

## 6. Conclusions

In this work, we examined cross-protocol domain shift when transferring an IP-trained IoT intrusion/anomaly detector to BLE traffic under target-unlabeled UDA constraints. Across leakage-aware random-window splits and a stricter capture-wise LOCO protocol, we found that reported UDA gains can be split-dependent: modest improvements under random splits can vanish (or reverse) under LOCO, where target ranking approaches chance, and simple baselines may dominate. Therefore, we treat LOCO evaluation, group-wise splitting, and operating-point audits as first-class requirements for deployment-faithful reporting, rather than optional stress tests.

We also show that domain-adversarial training is highly sensitive to checkpoint selection because domain confusion is transient. Over 20 seeds, DANN (default) does not provide statistically significant gains over ERM, while our domain-aware R3 rule yields more consistent AP improvements without using target labels for selection (Wilcoxon *p* < 0.05 for AP), although ROC-AUC gains are positive but not statistically significant. Finally, threshold transfer from the source domain can produce unsafe decisions (micro-FPR = 1.0 on benign-only captures), unless calibration and PR-oriented operating points are explicitly audited. Taken together, this study contributes both (i) an empirical account of when lightweight UDA succeeds or fails in cross-protocol IoT settings and (ii) a practical diagnostic workflow (Table 6) and reproducibility package to support robust, risk-aware model deployment.

## Figures and Tables

**Table 1:** Dataset statistics for Internet Protocol (IP, source) and Bluetooth Low Energy (BLE, target) after windowing (window size = 64; stride = 64).

Domain	Windows	y = 0	y = 1	Pos. Ratio
IP (source)	15,625	7813	7812	0.500
BLE (target)	3041	1273	1768	0.581

**Table 2:** Data splits for the representative run (seed = 2026). The target split is unlabeled during training and used only for alignment/diagnostics; labels are used solely for final reporting. The feature dimension is 14.

Domain	Train	Unlabeled Split	Test
Source (IP)	10,937	2344	2344
Target (BLE)	NA	1520	1521

Note: Target-domain class labels are used only for evaluation. NA: not applicable (no labeled target training split under UDA). The unlabeled target split is used as unlabeled data for alignment/diagnostics (e.g., CORAL/MMD/DANN losses). For the default early stopping baseline, checkpoints are selected using source validation performance only; our proposed R3 selection additionally uses domain labels (source vs. target, not class labels) on this unlabeled split to compute balanced domain accuracy (DomAcc) for checkpoint selection.

**Table 3:** Key architecture and training settings used in all experiments (unless stated otherwise). A complete environment snapshot is provided in Supplementary File S1. Abbreviations: ERM, empirical risk minimization; CORAL, correlation alignment; MMD, maximum mean discrepancy; DANN, domain-adversarial neural network; GRL, gradient reversal layer; DomAcc, domain-classifier accuracy.

Parameter	Value
Input features	14 window-level statistical features (packet length and inter-arrival time statistics); window size = 64, stride = 64.
Standardization	StandardScaler fitted on source (IP) train split only; applied to all splits/domains.
Feature extractor (G)	MLP, 14 → 128 → 128 (ReLU).
Classifier head (C)	Linear 128 → 2 (binary classification).
Domain discriminator (D)	MLP, 128 → 64 → 2 logits.
Training length	20 epochs for all methods.
GRL schedule (DANN)	$\lambda_{d}$ Warm-up epochs 1–3: $\lambda_{d}$ = 0, then increases monotonically toward 1 (see dann_train_history.csv).
Checkpoint selection	Best checkpoint selected on labeled source validation ROC-AUC (tie-breakers: source-validation AP; then earliest epoch); no target labels used.
Seeds	2024/2025/2026; seed controls data splitting, initialization, and minibatch order (Python/NumPy/PyTorch RNGs).
Optimizer	Adam (PyTorch), default $\beta_{1}$ = 0.9, $\beta_{2}$ = 0.999.
Learning rate	1 × 10^−3^.
Batch size	512.
Weight decay	0.0.
Dropout	Included in implementation (*p* = 0.0); see checkpoints/state_dict keys.
DomAcc evaluation set	Computed on a balanced domain-validation set formed by subsampling equal numbers from the source-validation and unlabeled target splits each epoch; therefore, chance = 0.5.
CORAL weight ($\lambda_{c}$)	1.0 (implicit; CORAL uses closed-form covariance alignment, no extra scaling term).
MMD weight (β(ep, step))	β(ep, step) schedule (MMD-ERM): loss = L_cls + β(ep, step)·L_mmd with EPOCHS = 20, WARMUP_EPOCHS = 3, β_max = 1.0. β(ep, step) = 0 for ep ≤ 3; else β(ep, step) = β_max·clip(*p*, 0, 1), where *p* = ((ep − WARMUP_EPOCHS − 1)·n_steps + step)/((EPOCHS − WARMUP_EPOCHS)·n_steps).
MMD kernel/bandwidth	Multi-kernel RBF MMD with kernel_mul = 2.0 and kernel_num = 5. Bandwidth normalization: bw = bw/${kernel\_mul}^{\left(kernel\_num//2\right)}$; bandwidth_list = bw∗${kernel\_mul}^{i}$(i = 0 to 4).

**Table 4:** Cross-protocol target BLE performance (mean ± std over seeds 2024/2025/2026). Classical ML baselines (LogReg/RF/XGB) are also reported. All results use active-window filtering (zero-activity windows removed); F1 and DomAcc_last are not applicable (NA) for these non-adversarial models. Note: F1@ can be inflated when a source-selected threshold transfers poorly to the target; therefore, we treat ROC-AUC/AP as primary metrics. Abbreviations: BLE, Bluetooth Low Energy; ERM, empirical risk minimization; CORAL, correlation alignment; MMD, maximum mean discrepancy; DANN, domain-adversarial neural network; GRL, gradient reversal layer; AP, average precision; DomAcc_last, final-epoch domain-classifier accuracy; LogReg, logistic regression; RF, random forest; XGB, XGBoost.

Method	Tgt AUC	Tgt AP	Tgt F1	DomAcc_last
CORAL-ERM	0.555 ± 0.094	0.712 ± 0.060	0.620 ± 0.030	NA
DANN (GRL)	0.620 ± 0.043	0.668 ± 0.046	0.613 ± 0.211	0.973 ± 0.002
ERM (source only)	0.590 ± 0.097	0.632 ± 0.057	0.735 ± 0.000	NA
MMD-ERM	0.613 ± 0.062	0.655 ± 0.034	0.614 ± 0.210	NA
noGRL (lambda = 0)	0.579 ± 0.079	0.625 ± 0.045	0.735 ± 0.000	0.996 ± 0.002
LogReg	0.683 ± 0.027	0.756 ± 0.078	NA	NA
RF	0.758 ± 0.051	0.770 ± 0.062	NA	NA
XGB	0.711 ± 0.013	0.706 ± 0.009	NA	NA

DomAcc_last is the final-epoch balanced domain accuracy of the domain classifier computed on a balanced domain-validation set (equal samples per domain); values near 0.5 indicate domain confusion.

**Table 5:** Bootstrap 95% confidence intervals on the BLE target test set (seed = 2026).

Method	AUC	AUC 95% CI	AP	AP 95% CI
ERM (source only)	0.501	[0.500, 0.502]	0.581	[0.559, 0.606]
noGRL (lambda = 0)	0.502	[0.500, 0.504]	0.583	[0.557, 0.608]
DANN (GRL)	0.626	[0.597, 0.653]	0.713	[0.680, 0.743]
Δ (DANN–ERM)	0.124	[0.096, 0.153]	0.131	[0.106, 0.157]

**Table 6:** Unified diagnostic workflow for cross-protocol transfer under target-unlabeled UDA (seed = 2026 numbers shown where applicable).

Stage/Question	Proxy Diagnostic (No Target Labels)	Interpretation and Recommended Action	Where It Is Reported
Split realism and leakage risk	Random window split vs. capture-wise/LOCO; group-wise splitting when IDs are available	Treat random splits as optimistic; always report capture-wise/LOCO results for deployment-faithful claims.	Section 3.3 and Section 4.7; Table 13
Global representation gap	SWD and kernel MMD (with σ sweep); DomAcc curves during adversarial training	Use multiple, complementary proxies; divergences can disagree (seed = 2026: SWD 66.115 → 40.679, MMD 0.579 → 0.669). Avoid final-epoch conclusions when DomAcc returns to ≈1.0.	Tables 7 and 8; Figures 3, 6 and 7
Within-class semantic shift	Class-conditional KS tests (per-feature, per-class)	Large within-class shifts suggest protocol/capture artifacts and can explain LOCO collapse despite apparent ‘alignment’.	Table 9; Section 4.7
Checkpoint selection without target labels	R3: choose a ‘star’ epoch among near-best source-validation epochs by preferring higher domain confusion (DomAcc closer to 0.5)	Mitigates seed-to-seed variance without using target labels. Report δ sensitivity and the oracle gap for context.	Section 4.5; Tables 10 and 11; Supplementary Table S1
Operating-point safety	Threshold-transfer audit (τ_F1 vs. τ at calibrated FPR); micro-FPR on benign-only captures; calibration checks	Prefer PR/AP and calibrated operating points; a source-tuned τ_F1 can yield unsafe micro-FPR = 1.0 under shift.	Section 4.6; Table 12; Figures 10–12
Reproducibility and auditability	Release derived features, split definitions, and scripts; report sensitivity analyses	Enable independent reproduction and auditing even under restricted raw pcap access; reduce single-author interpretation bias.	Supplementary File S1; Appendix A.5

**Table 7:** Domain-gap diagnostics (maximum mean discrepancy (MMD) and sliced Wasserstein distance (SWD)) before vs. after adaptation (seed = 2026).

Metric	Before	After
MMD	0.579	0.669
SWD	66.115	40.679

**Table 8:** MMD kernel bandwidth sensitivity (σ sweep) on 14D window-level feature vectors (IP vs. BLE; subsampled windows per domain). σ denotes the RBF kernel bandwidth; MMD^2^ is estimated with an unbiased estimator.

σ	MMD^2^ (Unbiased)
0.1	0.692760
0.2	0.723636
0.5	0.840080
1	1.040332
2	1.110997
5	0.438673
10	0.138348

**Table 9:** Semantic-shift diagnostic via class-conditional KS tests (IP vs. BLE). For each feature, we report KS statistics (D) and *p*-values within benign and attack subsets. Very small *p*-values may underflow to 0 in double precision; we report them as <1 × 10^−300^ for readability.

Feature	KS D (Benign)	*p* (Benign)	KS D (Attack)	*p* (Attack)
pkt_len_mean	0.556167	<1 × 10^−300^	1.000000	<1 × 10^−300^
pkt_len_min	0.966222	<1 × 10^−300^	1.000000	<1 × 10^−300^
pkt_len_median	0.587588	<1 × 10^−300^	1.000000	<1 × 10^−300^
iat_max	0.998429	<1 × 10^−300^	0.729937	<1 × 10^−300^
iat_std	0.998429	<1 × 10^−300^	0.729681	<1 × 10^−300^
iat_min	0.998429	<1 × 10^−300^	0.729372	<1 × 10^−300^
pkt_len_std	0.998301	<1 × 10^−300^	0.640511	<1 × 10^−300^
pkt_len_max	0.995159	<1 × 10^−300^	0.494227	<1 × 10^−300^
nonzero_ratio	0.966094	<1 × 10^−300^	0.625192	<1 × 10^−300^

**Table 10:** Domain-aware checkpoint selection (R3) under the active protocol. Per seed comparison between default-best (source-validation ROC-AUC) and domain-aware star (DomAcc closest to 0.5 among near-best epochs).

Seed	Best Epoch	Star Epoch	Tgt ROC-AUC (Best)	Tgt ROC-AUC (Star)	Delta ROC-AUC	Tgt AP (Best)	Tgt AP (Star)	Delta AP
2024	1	11	0.504	0.538	0.034	0.570	0.554	−0.015
2025	1	12	0.496	0.542	0.046	0.561	0.555	−0.006
2026	1	20	0.475	0.556	0.081	0.547	0.555	0.008
Mean ± Std			0.492 ± 0.015	0.545 ± 0.009	0.053 ± 0.024	0.559 ± 0.011	0.555 ± 0.000	−0.005 ± 0.012

**Table 11:** Oracle comparison for checkpoint selection (analysis-only upper bound) under the active protocol. Oracle epoch maximizes target-test ROC-AUC post hoc.

Seed	Best Epoch	Star Epoch	Oracle Epoch	Tgt ROC-AUC (Best)	Tgt ROC-AUC (Star)	Tgt ROC-AUC (Oracle)	Oracle Gap (Oracle − Star)
2024	1	11	20	0.504	0.538	0.542	0.004
2025	1	12	20	0.496	0.542	0.557	0.015
2026	1	20	17	0.475	0.556	0.556	0.000

**Table 12:** Operating point and calibration audit under leakage control (seed = 2026). AUC/AP are reported only for the mixed-class BLE capture group (ble_cap_2). Pos. ratio is the positive-class prevalence in ble_cap_2; AP-lift = AP − pos_ratio to contextualize AP under class imbalance. micro-FPR is computed across all capture groups (ble_cap_0/1/2) using negative counts; F1 is maximized on source validation, and (FPR = 1%) is the source-validation threshold, achieving 1% FPR on negatives. Abbreviations: AUC, area under the ROC curve; AP, average precision; FPR, false-positive rate; ECE, expected calibration error.

Model	AUC (ble_cap_2)	AP (ble_cap_2)	Pos. Ratio (ble_cap_2)	AP-Lift	Micro-FPR@$\tau^{*}$	Mi-cro-FPR@τ (FPR = 1%)	ECE (src − val)	ECE (tgt ble_cap_2)
ERM_MLP	0.501	0.714	0.714	0.000	1.000	1.000	0.577	0.286
DANN_GRL	0.501	0.714	0.714	0.000	0.684	0.684	0.562	0.286

**Table 13:** Leakage-controlled leave-one-capture-out (LOCO) performance on the only mixed-class BLE capture group (ble_cap_2). Mean ± std across seeds {2024, 2025, 2026}. AP-lift is defined as AP − pos_ratio to contextualize AP under class imbalance (pos_ratio = 0.714 for ble_cap_2).

Model	AUC	AP	AP-Lift
LogReg	0.670 ± 0.001	0.862 ± 0.001	0.148 ± 0.001
RF	0.593 ± 0.016	0.808 ± 0.004	0.094 ± 0.004
XGBoost	0.490 ± 0.067	0.713 ± 0.029	−0.001 ± 0.029
ERM_MLP	0.501 ± 0.000	0.714 ± 0.000	0.000 ± 0.000
DANN_GRL	0.497 ± 0.006	0.713 ± 0.002	−0.001 ± 0.002

**Figure A1:**
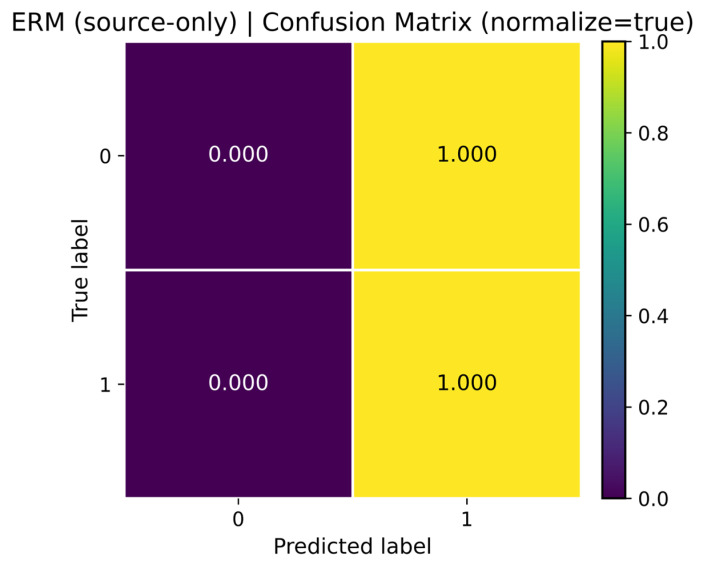
Normalized confusion matrix for ERM (source only) on the BLE target test set (seed = 2026).

**Figure A2:**
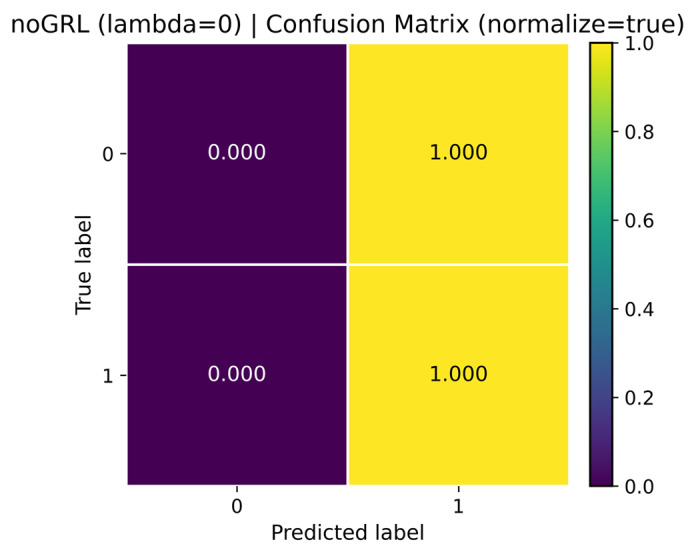
Normalized confusion matrix for noGRL (lambda = 0) on the BLE target test set (seed = 2026).

**Figure A3:**
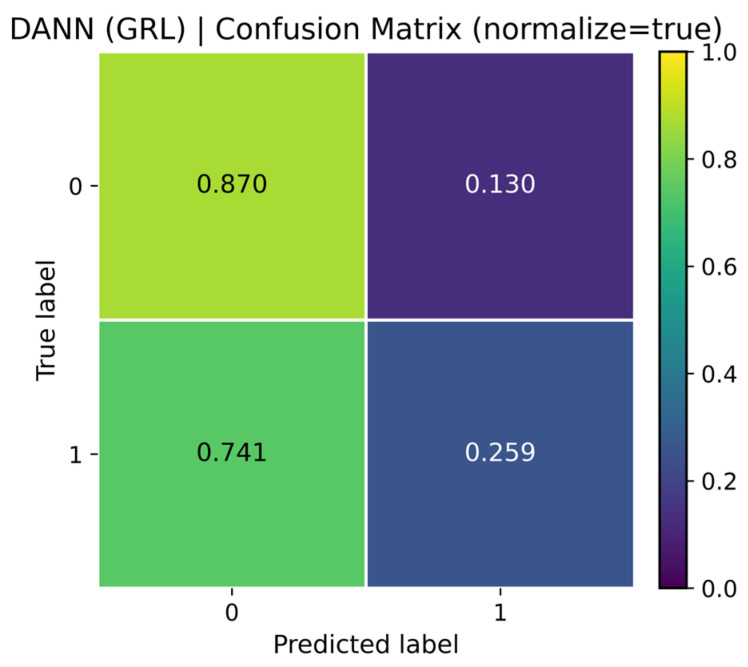
Normalized confusion matrix for DANN (GRL) on the BLE target test set (seed = 2026).

## Data Availability

The derived anonymized window-level feature tables (IP and BLE) and split definitions used in this study are provided as Supplementary Materials. Raw packet captures (pcap) contain sensitive network traces and are not publicly available due to privacy and security considerations; access may be granted for audit purposes upon reasonable request to the corresponding author. This study did not lead to any patents.

## Supplementary Materials

The following supporting information can be downloaded at: https://www.mdpi.com/article/10.3390/s26041184/s1, Table S1: δ-sensitivity analysis of R3 checkpoint selection. Supplementary File S1 (S1_repro_package.zip) contains the derived window-level feature tables (IP/BLE), split definitions, training logs, checkpoints, and scripts to reproduce the reported tables and figures. Supplementary File S2 (S2_additional_seeds_20seeds_summary.zip) provides additional seed repeats (20 seeds) and paired statistical tests supporting the instability analysis. Supplementary File S3 (S3_ip2zigbee_protocol_extension_summary.zip) provides the IP → Zigbee addendum (dataset notes and per seed results). Supplementary File S4 (addendum_v8_optional.zip) provides optional extended diagnostics and SCIE-grade schematics (e.g., proxy A-distance, MCD comparison, pipeline diagrams). Additional referenced artifacts: semantic_shift_ks_top.csv; mmd_sigma_sweep.csv; seed_sweep_classical_20seeds.csv; Figures.zip; tables.zip; FigureFilename.csv. Supplementary File S5 (sensors-26-01184_R1_SuppAddendum_SelfTraining20_S5.zip): 20-seed pseudo-label self-training addendum (Tables S2–S5; Figures S1–S4). Table S2: Self-training vs. ERM summary (random-window setting, 20 seeds). Table S3: Paired Wilcoxon tests (20 seeds). Table S4: Benign-only micro-FPR summary. Table S5: Pseudo-label counts. Figures S1–S4: Paired AUROC/AP and delta distributions (random-window setting).

## Abbreviations

AP, average precision; AP-lift, AP-pos_ratio (positive prevalence); AUC, area under the curve; BLE, Bluetooth Low Energy; CORAL, correlation alignment; DANN, domain-adversarial neural network; DomAcc, domain-classifier accuracy; ECE, expected calibration error; ERM, empirical risk minimization; FPR, false positive rate; GRL, gradient reversal layer; IDS, intrusion detection system; IoT, Internet of Things; LOCO, leave one capture out; LogReg, logistic regression; MLP, multilayer perceptron; MMD, maximum mean discrepancy; PR, precision–recall; RF, random forest; ROC-AUC, receiver operating characteristic area under the curve; SWD, sliced Wasserstein distance; t-SNE, t-distributed stochastic neighbor embedding; UDA, unsupervised domain adaptation; XGB, XGBoost; Zigbee, IEEE 802.15.4-based low-power wireless protocol; ZBDS, Zigbee dataset (ZBDS2023).

## Appendix A

### Appendix A.1. Confusion Matrices (Seed 2026)

For completeness, Figure A1, Figure A2 and Figure A3 show normalized confusion matrices on the BLE target test set (seed = 2026) for representative methods.

### Appendix A.2. Thresholded Error Analysis (Seed 2026)

As summarized in Table A1, we report thresholded confusion counts and class-wise performance on the BLE target domain for the seed = 2026 runs.

**Table A1:** Thresholded confusion counts and class-wise performance on the BLE target (seed = 2026).

Method	$\tau^{*}$ (src − val)	TN	FP	FN	TP	Prec_pos	Rec_pos	F1_pos	Acc
ERM	0.388	0	637	0	884	0.581	1.000	0.735	0.581
noGRL (lambda = 0)	0.381	0	637	0	884	0.581	1.000	0.735	0.581
DANN (GRL)	0.271	554	83	655	229	0.734	0.259	0.383	0.515

$\tau^{*}$ is chosen by maximizing the attack-class F1 on the source validation split and then applied unchanged to the BLE target test set. TN/FP/FN/TP are computed on the BLE target domain with class 1 = attack.

### Appendix A.3. Implementation Details and Hyperparameters

In Table A2, we summarize the model architectures, training schedules, and other implementation details used to reproduce the reported metrics without accessing restricted raw packet traces.

**Table A2:** Implementation details and hyperparameters.

Parameter	Value
Input features	14 window-level statistical features (packet length and inter-arrival time statistics); window size = 64, stride = 64.
Feature standardization	StandardScaler fitted on source (IP) training split only; applied to all splits/domains.
Feature extractor (G)	MLP, 14 → 128 → 128 (ReLU).
Classifier head (C)	Linear 128 → 2 (binary classification).
Domain discriminator (D)	MLP, 128 → 64 → 2 logits.
Training epochs	20 epochs for all methods.
DANN objective	Equation (4) (implemented via GRL); domain loss is CE on domain logits.
GRL schedule	$\lambda_{d}$ Warm-up epochs 1–3: $\lambda_{d}\;=\;0$, then increases monotonically toward 1 (see dann_train_history.csv).
Default best checkpoint	Selected using labeled source validation only (maximize source-validation ROC-AUC; tie-breakers: source-validation AP; then earliest epoch).
Epoch (diagnostic)	Diagnostic epoch defined as the epoch where domain accuracy is closest to 0.5 (maximal domain confusion).
Random seeds	2024/2025/2026; seed controls split shuffling, model initialization, and minibatch order (Python/NumPy/PyTorch RNGs).
Environment snapshot	Python 3.10.12; PyTorch 2.7.1 + cu118; scikit-learn 1.7.2; GPU: NVIDIA A100 80 GB; full package list in Supplementary File S1 (Supplementary S1).
Optimizer	Adam (PyTorch), default $\beta_{1}\;=\;0.9,\;\beta_{2}$ = 0.999.
Learning rate	1 × 10^−3^.
Batch size	512.
Weight decay	0.0.
Dropout	Included in implementation (*p* = 0.0); see checkpoints/state_dict keys.
DomAcc evaluation set	Computed on a balanced domain-validation set formed by subsampling equal numbers from the source-validation and unlabeled target splits each epoch; therefore, chance = 0.5.
CORAL/MMD weights	Alignment weights follow the released implementation in S1 (see scripts/config).
MMD kernel bandwidth	RBF kernel; bandwidth selection is documented in S1 (scripts/config).
$\mathrm{DANN}\;\lambda_{d}$ schedule	$\lambda_{d}$ Warm-up epochs 1–3: $\lambda_{d}$ = 0; then, monotonic increase toward 1 (logistic schedule; see logs/dann_train_history.csv).
$\mathrm{CORAL}\;\mathrm{weight}\;(\lambda_{c}$)	1.0 (implicit; CORAL uses closed-form covariance alignment, no extra scaling term).
MMD weight (β(ep, step))	β(ep, step) schedule (MMD-ERM): loss = L_cls + β(ep, step)·L_mmd with EPOCHS = 20, WARMUP_EPOCHS = 3, β_max = 1.0. β(ep, step) = 0 for ep ≤ 3; else β(ep, step) = β_max·clip(*p*, 0, 1), where *p* = ((ep − WARMUP_EPOCHS − 1)·n_steps + step)/((EPOCHS − WARMUP_EPOCHS)·n_steps).
MMD RBF bandwidth	Bandwidth selection rule documented in S1/scripts/config (RBF kernel).
MMD kernel/bandwidth	Multi-kernel RBF MMD with kernel_mul = 2.0 and ker-nel_num = 5. Bandwidth normalization: bw = bw/${kernel\_mul}^{\left(kernel\_num//2\right)}$; bandwidth_list = bw * ${kernel\_mul}^{i}$ (i = 0 to 4).

### Appendix A.4. Seed-Robust Analyses (20 Seeds)

Seed-robust classical baselines on the BLE target test set are summarized in Table A3, and corresponding paired tests are reported in the same subsection.

**Table A3:** Seed-robust classical baselines on the BLE target test set (20 seeds, 2024–2043). Paired Wilcoxon tests: RF-LR *p* = 1.91 × 10^−6^ (AUC/AP); XGB-RF *p* = 0.0027 (AUC/AP).

Model	ROC-AUC (Mean ± Std)	AP (Mean ± Std)
Logistic Regression (LR)	0.6456 ± 0.0151	0.6352 ± 0.0159
Random Forest (RF)	0.7560 ± 0.0428	0.7669 ± 0.0538
XGBoost (XGB)	0.7137 ± 0.0184	0.7057 ± 0.0150

**Table A4:** ERM vs. DANN (default) vs. R3 across 20 seeds (2024–2043) under the target-unlabeled constraint. Paired Wilcoxon tests: DANN-ERM *p* = 0.498 (AUC), 0.674 (AP); R3-DANN *p* = 0.1769 (AUC), 0.0153 (AP); R3-ERM *p* = 0.1231 (AUC), 0.0172 (AP).

Method	ROC-AUC (Mean ± Std)	AP (Mean ± Std)
ERM	0.5909 ± 0.0383	0.6178 ± 0.0270
DANN (default)	0.5989 ± 0.0366	0.6216 ± 0.0277
R3 (checkpoint selection)	0.6376 ± 0.1253	0.6706 ± 0.0771

### Appendix A.5. Provenance and Data Usage Map

**Table A5:** Structured provenance summary for the released feature tables (sanitized). Raw packet traces are restricted; we release derived window-level features and column mappings.

Domain	Raw Packet Table (Sanitized CSV)	Time Column	Length Column	Label Column	Windowing
IP (source)	IP-Based Packets Preprocessed Dataset.csv	http.time	http.content_length	is_malicious	W = 64, stride = 64
BLE (target)	BLE Preprocessed Dataset.csv	nordic_ble.packet_time	frame.len	is_malicious	W = 64, stride = 64

**Table A6:** Data-usage map under the target-unlabeled constraint (what is used where).

Purpose	Source (IP)	Target (BLE)	Target Class Labels?
Alignment training	Train split	Unlabeled split	No
Checkpoint selection (R3/DomAcc)	Validation split (balanced)	Unlabeled split (balanced)	No
Final reporting	Test split	Test split	Yes (reporting only)

## Appendix B

### Protocol Pair Extension: IP → Zigbee (ZBDS2023)

We extend the protocol-pair analysis by evaluating IP → Zigbee transfer on the ZBDS2023 dataset. Zigbee captures were preprocessed with tshark/pyshark using the IEEE 802.15.4 FCS format set to “TI CC24xx metadata” (wpan.fcs_format) to recover timestamps. We form non-overlapping 64-frame windows and compute the same 14D window-level statistics used in the main study (packet-length and inter-arrival descriptors). Attack windows are labeled using the published attack-start list (types A–E) with a conservative fixed duration. The target Zigbee set contains 29,375 windows with an attack ratio of 0.0543 across 57 pcap groups. Full preprocessing notes and per-seed outputs are provided in Supplementary File S3.

The results of the additional protocol pair evaluation (IP → Zigbee, ZBDS2023) are summarized in Table A7.

**Table A7:** Additional protocol-pair evaluation (IP → Zigbee, ZBDS2023). Target ranking metrics (ROC-AUC/AP) reported as mean ± std over three seeds (2024/2025/2026).

Method	Target ROC-AUC (Mean ± Std)	Target AP (Mean ± Std)
ERM	0.500 ± 0.000	0.052 ± 0.025
DANN (default)	0.578 ± 0.135	0.200 ± 0.262
DANN (R3)	0.510 ± 0.010	0.065 ± 0.035

These addendum results reinforce the protocol-general message of the main study: sour-e only ERM can collapse to chance under cross-protocol shift, and adversarial alignment may improve target ranking in some runs but remains seed-sensitive. Therefore, we emphasize leakage-controlled evaluation and checkpoint/operating-point audits for cross-protocol deployment.
